# PD-L1 expression in anogenital and oropharyngeal squamous cell carcinomas associated with different clinicopathological features, HPV status and prognosis: a meta-analysis

**DOI:** 10.1042/BSR20203669

**Published:** 2021-03-26

**Authors:** Yuan Qin, Jiaochen Luan, Xiang Zhou, Ying Li

**Affiliations:** 1 Department of Urology, The Second Affiliated Hospital of Nanjing University of Chinese Medicine, Jiangsu Provincial Second Chinese Medicine Hospital, Nanjing, Jiangsu, China; 2 Department of Urology, The First Affiliated Hospital of Nanjing Medical University, Nanjing, Jiangsu, China; 3 Department of Oncology, The Second Hospital of Nanjing, Nanjing University of Chinese Medicine, Nanjing, Jiangsu, China

**Keywords:** clinicopathology, HPV, meta-analysis, PD-L1, squamous cell carcinoma

## Abstract

**Background:** Little research has been done on clinicopathological characteristics and human papillomavirus (HPV) status of anogenital and oropharyngeal squamous cell carcinomas (SCC) with a strong expression of programmed death ligand 1 (PD-L1) in tumor cells. Therefore, we conducted this meta-analysis. **Methods:** We performed a comprehensive research in PubMed, Embase and Cochrane databases up to 30 September 2020. The effect size was hazard ratio (HR) with 95% confidence interval (CI) for overall survival (OS), cancer-specific survival (CSS), disease-free survival (DFS). The pooled odds ratio (OR) with 95% CI were used to assess the association between PD-L1 expression and clinicopathological features along with HPV status. **Results:** A total of 2003 cases (944 anogenital and 1059 oropharynx SCC patients) were included. High PD-L1 expression in anogenital SCC cases were associated with advanced age (OR = 1.63, 95% CI: 1.04–2.58) and HPV negativity (OR = 0.47, 95% CI: 0.31–0.71). Besides, PD-L1 positive anogenital SCC cases held a significantly declined OS (HR = 2.18, 95% CI: 1.37–3.47) and CSS (HR = 2.45, 95% CI: 1.30–4.65). For oropharynx SCC, PD-L1 was more frequent in younger and HPV positive patients (OR = 0.60, 95% CI: 0.37–0.98; OR = 3.01, 95% CI: 1.78–5.09) and PD-L1 expression was relevant to better OS and DFS (HR = 0.76, 95% CI: 0.60–0.97; HR = 0.50, 95% CI: 0.33–0.75). **Conclusions:** The meta-analysis demonstrated that in anogenital SCC, PD-L1 positivity had to do with a worse outcome, which might attribute to advanced age, higher tumor grade, lymph node metastasis and HPV negativity, while in oropharynx cancer, PD-L1 expression was related to better prognosis for the reason that PD-L1 was less frequent in the aged and negative HPV status.

## Introduction

Human papillomavirus (HPV)-related cancers account for 8.6% of female and 0.8% of male carcinomas globally, and persistent high-risk HPVs infection is the fundamental reason. High-risk HPVs not only contribute to more than 80% of cervical cancer cases, but also take responsibility for approximately 88% of anal, 78% of vaginal, 51% of penile, 40% of vulvar and 30% of oropharynx cancer cases. Besides, these malignancies account for almost all of HPV-related cancers [1]. Thanks to promoting screening programs and advances in treatment of cervical lesion, the incidence and mortality rates of cervical cancer generally have declined over the last decade. However, this tendency varies in different countries and regions [2]. Among men, approx. 3200 new anal cancer along with 26000 new penile cancer cases occur annually. Meanwhile, approximately 4850 new anal cancer in 2016, 6190 new vulvar cancer in 2018 and 12000 vaginal cancer cases in 2012 were diagnosed among women worldwide [1,3,4].

In addition to anogenital cancers, head and neck cancer is the sixth leading cancer worldwide with 600000 new cases per year and a mortality rate of approx. 450000 per year, occupying 78% of HPV-related oropharynx cancer cases [1,5]. Meanwhile, the HPV positive oropharyngeal cancers have been increasing during the last decade, even though the consumption of tobacco declining, especially among youngsters in more developed countries [1]. Overall, early-stage HPV-related cancer patients have an optimal outcome with a high probability of cure. However, patients with advanced cancers, particularly with regional lymph nodes and distant metastases, may undergo multimodal treatments, including lymphadenectomy and chemoradiotherapy [6]. Unfortunately, these aggressive strategies can not prevent patients with high-stage carcinomas from treatment failure and poor prognosis. Therefore, new effective and optimized therapeutic options for those with advanced tumors are urgent.

Recently, the early- and late-phase clinical researches show that the efficiency of immunotherapies in certain solid tumors brings hope for patients, such as anti-programmed death 1 (PD-1)/programmed death ligand 1 (PD-L1) immunotherapy, which is one of the significant immunotherapies. And, PD-1 or PD-L1 blockade therapy is more effective than conventional therapies in advance solid tumors [7]. PD-1, a co-inhibitory receptor existing on T cells, B cells and natural killer (NK) cells, is combined with its ligand PD-L1 in tumor cells, which suppresses the activation and proliferation of T cells. As a result, tumor cells can escape from host anti-tumor immunity. By stopping the PD-1/PD-L1 interacting with PD-1 or PD-L1 blockade therapy, cytotoxic T cells are re-stimulated to eliminate cancer cells [8]. There are many kinds of cancers expressing PD-L1, including HPV-related malignancies.

The studies investigating the relationship between PD-L1 expression in tumor cells and clinical features in anogenital and oropharyngeal squamous cell carcinomas (SCCs) are growing [9–29], which could identify potential pathological characteristics for judging whether patients could get clinical benefits from PD-1 or PD-L1 blockade therapy with variable PD-L1 expression and investigate the role of PD-L1 in anogenital and oropharyngeal SCC. However, the associations between PD-L1 expression in anogenital and oropharyngeal SCC and clinicopathological features, HPV status (i.e. based on HPV DNA, p16 overexpression or both [30]) and prognosis are inconsistent. Therefore, we conducted the meta-analysis to clarify the association between PD-L1 expression in cancer cells and outcome, and to assess the relationship between PD-L1 expression in tumor cells and clinicopathological features along with HPV status in anogenital and oropharyngeal SCC.

## Methods

### Literature search

A comprehensive research was conducted in PubMed, Embase and Cochrane databases based on ‘PRISMA’ guidelines up to 30 September 2020. Furthermore, there was no language restriction. The following items were used for searching: (‘programmed cell ligand 1’ or ‘PD-L1’ or ‘B7-H1’ or ‘CD274’) and (‘HPV-related’ or ‘penile’ or ‘penis’ or ‘cervical’ or ‘cervix’ or ‘vaginal’ or ‘vagina’ or ‘vulvar’ or ‘vulva’ or ‘anal’ or ‘anus’ or ‘oropharyngeal’ or ‘base of tongue’ or ‘posterior pharyngeal wall’ or ‘tonsil’ or ‘soft palate’) and (‘cancer’ or ‘tumor’ or ‘neoplasm’). Furthermore, the references of included articles were also manually screened for other potential eligible researches.

### Inclusion and exclusion criteria

Eligible studies had to fulfill the following criteria: (1) all SCC were diagnosed by pathology results. (2) PD-L1 expression was identified in tumor cells by immunohistochemistry (IHC) staining. (3) The associations between PD-L1 expression and clinicopathological features, prognosis or HPV status were demonstrated in study. (4) Both HPV positive and negative patients were included in the studies. The reasons why studies were excluded were as follows: (1) no data available could be extracted from papers. (2) Studies illustrated the role of PD-L1 expressing in other cells in cancer tissues or non-SCC. (3) Patients were treated with PD-1 or PD-L1 blockade therapy. (4) Studies were non-original researches. (5) Studies only contained HPV positive or negative patients. Additionally, when the same patients occurred or were mixed in different studies, only the most recent and complete studies were included.

### Data extraction

The relevant data were extracted from eligible studies by two independent authors (X.Z.) and (J.c.L.), which were examined by other investigators (Y.L. and Y.Q.). The data extracted from the eligible studies were recorded as follows: first author, year of publication, cancer type, country of patients population, gender, age, follow-up period, patients sample size, pathology, methods for PD-L1 detection, PD-L1 distribution, cut-off value of PD-L1 positive expression, PD-L1 positive expression rate, tumor stage and outcomes of patients, including overall survival (OS), cancer-specific survival (CSS), disease-free survival (DFS) with hazard ratios (HRs) with 95% confidence interval (CI). Moreover, Newcastle–Ottawa Scale was applied to assess the quality of included papers.

### Statistical analysis

Stata software (version 12.0; Stata Corp LP, College Station, TX) was applied for present meta-analysis. The relationships between PD-L1 and clinicopathological characteristics along with HPV status were evaluated by odds ratio (OR) with 95% CI. If studies provided HPV-DNA and P16 status in papers, we considered that P16 was a superior biomarker to HPV-DNA due to P16 best representing oncogenic activity of the HPV in tumors [31]. HR with 95% CI directly reported in eligible studies was applied to assess the role of PD-L1 in prognosis of patients, and we also adopted method described by Altman and Bland to calculate 95% CI, when its HR and *P*-value were provided in study [32]. Moreover, if studies offered Kaplan–Meier curves rather than HR with 95% CI, we extrapolated HR with 95% CI by data directly obtained from the curves. When multivariable and univariable outcome analyses were offered, the former was adopted. Because of the obvious differences between anogenital cancers (cervical, vaginal, vulvar, penile and anal cancer) and oropharyngeal cancers in embryological origins, as well as unequivocal premalignant and malignant changes existing in entire anogenital region, we divided anogenital and oropharyngeal SCC in this meta-analysis into two main types, anogenital and oropharyngeal SCC, of which we performed meta-analysis, respectively.

Heterogeneity between researches was evaluated by Chi-square test and I-square test, and when value of *P*<0.10 or *I^2^* > 50%, we considered heterogeneity present in studies and utilized the random-effects model rather than fixed-effects model to calculate the pooled ORs/HRs. Otherwise, the fixed-effects model was applied with *P>*0.10 and *I^2^* < 50%. Additionally, we performed one-way sensitivity analysis to assess the stability of the results. Moreover, Egger’s and Begg’s tests were applied to evaluate the publication bias among the studies. Two-tailed *P*-values less than 0.05 were considered statistically significant.

## Results

### Literature search

**Figure 1:**
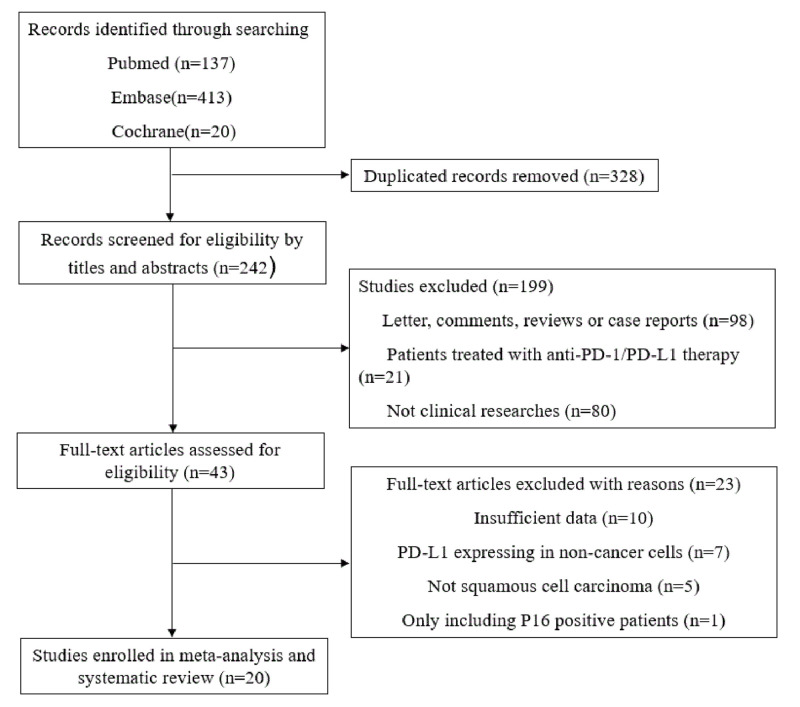
Study selection process

Initially, a sum of 570 articles were identified according to previous search strategy. Then, 328 duplications were excluded. After reviewing the title and abstract, 199 studies were removed for reasons. The full texts of the remaining articles were estimated, and 23 studies were further excluded. Eventually, we included 20 articles from the rest studies in total. A flow diagram detailing the selection process of studies was shown in Figure 1.

### Study characteristics

A total of 20 studies were screened out after comprehensive review and involved in the meta-analysis, which consisted of 2003 cases, including 944 anogenital SCC (246 cervical, 265 vulvar, 366 penile, 67 anal SCC cases) and 1059 oropharynx SCC patients [9–29]. All studies were retrospective and published between 2013 and 2020. IHC was applied to assess the expression of PD-L1 protein, and PD-L1 positivity was defined as percent of positive tumor cells in overall cancer cells with membranous or cytoplasmic staining. The commonest cut-off value of PD-L1 positivity was 5% in the meta-analysis [10,11,13,14,18,20,21,23,27–29]. Furthermore, there were three studies combining percentage of positive cells with staining intensity as a new definition of PD-L1 positivity (H-score) [12,17,24]. In addition, the relationships between HPV status and PD-L1 were investigated in eight studies of anogenital SCC [9–11,13,15,17,19,20], covering cervical, anal, penile and vulvar cancer cases, and eight oropharynx SCC studies [21–26,28,29]. Among these researches, HPV-DNA status was considered as the biomarker of HPV oncogenic activity and were used to assess their associations with PD-L1 expression in three anogenital and one oropharynx cancer studies [11,15,20,23]. Detailed characteristics of included studies are summarized in Tables 1 and 2.

**Table 1:** Detailed characteristics of the studies included in this meta-analysis

First author	Year	Cancer type	Country	Gender	Age (yr) (mean/median)	Follow-up (mo) (mean/median)	No.pts	Pathology	HPV detection method	PD-L1 distribution	Cut-off value	PDL1 pos percent	Tumor stage (T/N/M)	Outcome	NOS
Govindarajan, R.	2016	Anal cancer	U.S.A.	Male and female	52	NR	41	SCC	P16 (IHC)	mem/cyt	NR	56.10%	T1-T4/N0-N+/M0-M+	NR	7
Zhao, Y.J.	2018	Anal cancer	China	Male and female	52.5	40.9	26	SCC	P16 (IHC)	mem	5%	46.15%	T1-T4/N0-N+/NR	OS^SC^	8
Heeren, A.M.	2016	Cervical cancer	Netherlands	Female	46	NR	156	SCC	NR	mem/cyt	5%	40.38%	NR	NR	7
Wang, S.	2018	Cervical cancer	China	Female	46	61.05	90	SCC	NR	NR	H- score > 100	57.80%	T1-T2/N0-N+/NR	OS^U^	8
Udager, A.M.	2016	Penile cancer	U.S.A.	Male	63	NR	37	SCC	HPV-DNA	mem	5%	62.20%	T1-T4/N0-N+/M0-M+	CSS^U^,^1^	9
Deng, C.	2017	Penile cancer	China	Male	53	NR	116	SCC	NR	mem	5%	53.40%	T1-T4/N0-N+/NR	CSS^M^	9
Ottenhof, S.R.^2^	2018	Penile cancer	Netherlands	Male	65.9	100.7	213	SCC	HPV-DNA	mem	1%	48%	T1-T4/N0-N+/NR	CSS^M^	9
Howitt, B.E.	2016	Vulvar cancer	U.S.A.	Female	69	NR	23	SCC	IHC	mem/cyt	H-score > 100	39.10%	T1-T3/NR	NR	6
Sznurkowski, J.J.	2017	Vulvar cancer	Poland	Female	68	NR	84	SCC	P16	mem	5%	32.10%	T1-T4/NR	OS^U^	7
Hecking, T.	2017	Vulvar cancer	Germany	Female	64	46.7	103	SCC	SIH/P16	mem	9.70%	23.30%	T1-T4/N0-N+/M0-M+	OS^U^	8
Choschzick, M.	2018	Vulvar cancer	Switzerland	Female	68.9	NR	55	SCC	SIH	mem	5%	27.30%	T1-T4/N0-N+/NR	OS^NR^	6
Ukpo, O.C.	2013	Oropharynx cancer	U.S.A.	Male and female	55.8	NR	181	SCC	P16	mem+cyt	5%	46.40%	T1-T4/N0-N+/M0-M+	OS^U,a^	7
Kim, H.S.	2016	Oropharynx cancer	Korea	Male and female	57.5	44	133	SCC	P16	mem	20%	68%	T1-T4/N0-N+/M0	OS^U^	9
De Meulenaere, A.	2017	Oropharynx cancer	Belgium	Male and female	NR	NR	99	SCC	ISH	mem/cyt	5%	23%	T1-T4/N0-N+/M0	DFS^U^	8
Steuer, C.E.	2018	Oropharynx cancer	Georgia	Male and female	59	NR	97	SCC	P16	mem/cyt	H-score:1-130	25%	T1-T4/N0-N+/NR	OS^M^	8
Fukushima, Y.	2018	Oropharynx cancer	Japan	Male and female	NR	36	92	SCC	NR	mem/cyt	1%	75%	T1-T4/N0-N+/M0-M+	OS^M^	6
Hong, A.M.	2019	Oropharynx cancer	Australia	Male and female	59	NR	214	SCC	P16/HPV-DNA	mem	1%	67.8%	T1-T4/N0-N+/M0-M+	OS^M^	7
Sato, F.	2019	Oropharynx cancer	Japan	Male and female	63	37	137	SCC	NR	mem/cyt	5%	59.1%	T1-T4/N0-N+/M0-M+	OS^M^, DFS^M^	7
Lyford-Pike, S.	2013	Tonsil cancer	U.S.A.	NR	NR	NR	27	SCC	ISH/IHC	mem	5%	70%	NR	NR	6
Kwon, M.J.	2018	Tonsil cancer	Korea	Male and female	NR	NR	79	SCC	Chip test	mem/cyt	5%	29.10%	T1-T4/N0-N+/NR	OS^M^, DFS^M^	8

mo, month; yr, year; NR, not reported; No.pts, number of patients; U, univariate analysis; M, multivariate analysis; SC, survival curve; NOS, Newcastle–Ottawa Scale; ISH, *in situ* hybridization;

mem/cyt, PD-L1 positivity was defined as tumor cell membranous and/or cytoplasmic staining;

mem, PD-L1 positivity was defined as tumor cell membranous staining;

mem+cyt, PD-L1 positivity was defined as tumor cell membranous and cytoplasmic staining;

1 , 95% CI was calculated by method described by Altman and Bland [32], when its HR and *P*-value were provided in the study.

2 , Two studies shared this patient population. Only HR and 95% CI of CSS (diffuse vs negative/margin PD-L1 tumor-cell expression) were extracted from one study.

**Table 2:** Association between PD-L1 in tumor cells and clinicopathological characteristics in HPV-related SCC

Clinical parameters	Number of studies (number of patients)	OR (95% CI)	Model	Heterogeneity		Significance (*P*)
				*I^2^*	*P*	
Anogenital SCC						
Gender (male vs female)	2 (67)	0.32 (0.10–1.01)	Fixed	0.0%	0.979	0.052
Age (old vs young)^1^	5 (292)	1.63 (1.04–2.58)	Fixed	45.6%	0.118	0.035
T stage (T3/T4 vs T1/T2)	4 (397)	1.25 (0.74–2.11)	Fixed	20.1%	0.289	0.400
Grade (G3 vs G1/G2)	4(318)	2.49 (1.39–4.46)	Fixed	0.0%	0.790	0.002
Lymph node metastases (present vs absent)	7(627)	1.85 (1.28–2.66)	Fixed	32.9%	0.177	0.001
Distant metastases (present vs absent)	2(140)	5.31 (0.97–28.96)	Fixed	0.0%	0.414	0.054
Recurrence (present vs absent)	2(78)	2.48 (0.70–8.83)	Fixed	0.0%	0.739	0.161
Oropharynx SCC						
Gender (male vs female)	6(843)	0.39 (0.14–1.07)	Random	74.8%	0.001	0.067
Age (old vs young)^2^	3(349)	0.60 (0.37–0.98)	Fixed	17.5%	0.298	0.042
T stage (T3/T4 VS T1/T2)	6(843)	0.96 (0.71–1.30)	Fixed	7.6%	0.368	0.796
Grade (G3 VS G1/G2)	3(315)	3.40 (1.81–6.40)	Fixed	0.0%	0.389	0.000
Lymph node metastases (present vs absent)	5(706)	1.97 (1.32–2.92)	Fixed	30.5%	0.218	0.001

1 , Cut-off values of age included in meta-analysis were 52.5, 46, 63, 53 or 69 years, respectively.

2 , Cut-off values of age included in meta-analysis were 60, 63 or 65 years, respectively.

### PD-L1 expression and clinicopathological features

**Figure 2:**
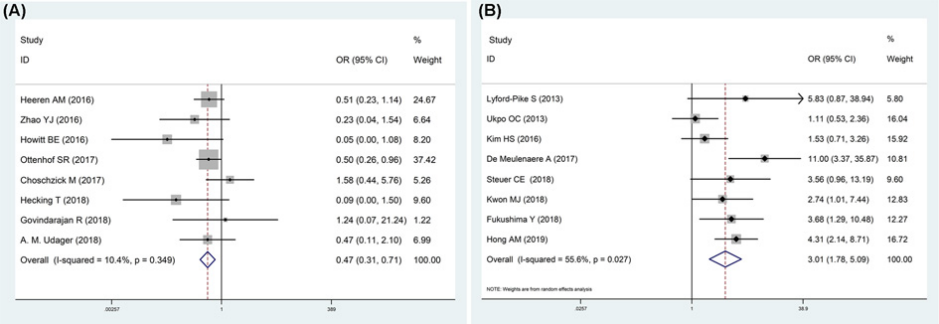
Forest plots for the association between PD-L1 expression and HPV status in anogenital and oropharyngeal SCC (**A**) Association between PD-L1 expression and HPV status in anogenital SCC. (**B**) Association between PD-L1 expression and HPV status in oropharyngeal SCC.

In anogenital SCC, advanced age, higher tumor grade and lymph node metastases were related to PD-L1 positive expression (OR = 1.63, 95% CI :1.04–2.58; OR = 2.49, 95% CI: 1.39–4.46; OR = 1.85, 95% CI: 1.28–2.66) (Table 2), demonstrated by the meta-analysis. However, no correlation was found among gender, tumor stage, recurrence or distant metastases and PD-L1 expression (Table 2). Contrary to anogenital SCC, PD-L1 in oropharynx SCC was less frequent in older patients (OR = 0.60, 95% CI: 0.37–0.98) (Table 2). Moreover, higher tumor grade and lymph node metastases were associated with PD-L1 positive expression in oropharynx SCC (OR = 3.40, 95% CI: 1.81–6.40; OR = 1.97, 95% CI: 1.32–2.92) (Table 2). The pooled OR of eight anogenital SCC studies showed that PD-L1 negative expression was connected with HPV positivity (OR = 0.47, 95% CI: 0.31–0.71, *P*=0.000) (Figure 2A) with a fixed-effects model, and there was low heterogeneity (*I^2^* = 10.4%, *P*=0.349) (Figure 2A). In order to reduce heterogeneous variables among the anogenital SCC studies, subgroups of meta-analysis were performed based on cancer types, antibody catalogs and cut-off values of PD-L1 positivity. The results demonstrated that there were significant differences in the relationship between PD-L1 expression and HPV status in the groups of penile SCC, PD-L1 antibody (clone E1L3N) and cut-off value greater than or equal to 5% (Supplementary Table S1).e main-text citation for Supplementary Table S1 is this paragraph.

However, PD-L1 expression was higher in HPV positive oropharynx SCC patients with moderate heterogeneity in a random-effects model (OR = 3.01, 95% CI: 1.78–5.09, *P*=0.00; *I^2^* = 55.6%, *P*=0.027) (Figure 2B). Stratified analysis showed that associations between high PD-L1 level and HPV positivity was significantly different in the group of antibody (clone SP142) and cut-off value greater or less than 5% (Supplementary Table S2).

### PD-L1 expression and oncological prognosis

**Figure 3:**
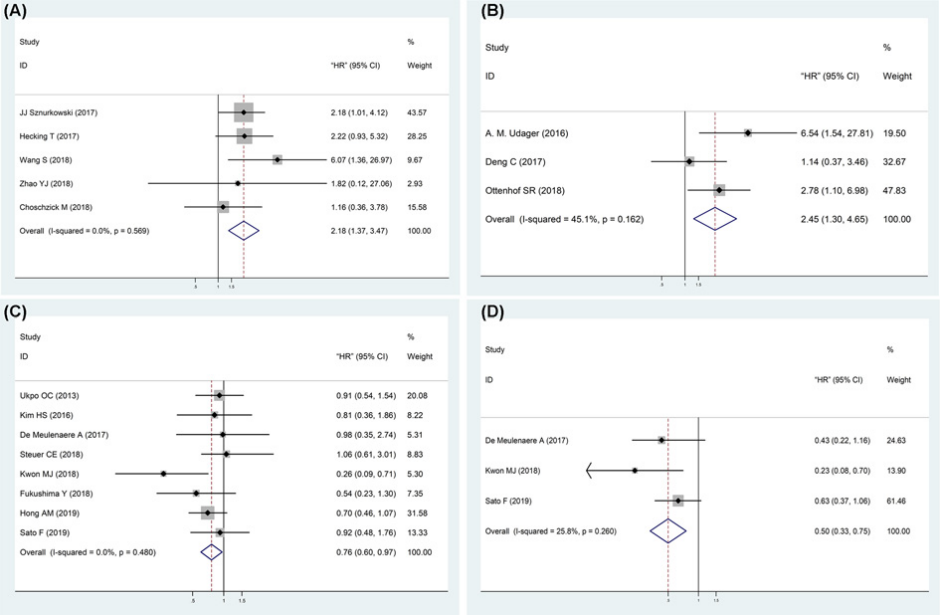
Forest plots for the association between PD-L1 expression and oncological prognosis in anogenital and oropharyngeal SCC (**A**) OS of anogenital SCC. (**B**) CSS of penile SCC. (**C**) OS of oropharyngeal SCC. (**D**) DFS of oropharyngeal SCC.

In general, anogenital SCC cases that were PD-L1 positive held a significantly declined OS compared with PD-L1 negative patients (HR = 2.18, 95% CI: 1.37–3.47, *P*=0.001; *I^2^* = 0.0%, *P*=0.569) (Figure 3A). Subgroup analysis according to cancer types, antibody catalogs and cut-off values of PD-L1 positivity were conducted, and our meta-analysis demonstrated that the predictive value of PD-L1 expression for OS of vulvar SCC, antibody (clone 22C3) and cut-off value greater than or equal to 5% (Supplementary Table S3). Our meta-analysis also suggested that PD-L1 positive penile SCC cases held a worse CSS (HR = 2.45, 95% CI: 1.30–4.65, *P*=0.006; *I^2^* = 45.1%, *P*=0.162) (Figure 3B).

In contrast with anogenital SCC, PD-L1 expression in oropharynx SCC was also a predictive value of OS (HR = 0.76, 95% CI: 0.60–0.97, *P*=0.025; *I*^*2*^ = 0.0%, *P*=0.480) (Figure 3C). Subgroup analysis was also conducted in accordance with antibody catalogs and cut-off values of PD-L1 positivity, and our meta-analysis suggested that the predictive value of PD-L1 expression for OS (clone SP142) and cut-off value were less than 5% (Supplementary Table S3). Meanwhile, PD-L1 expression was associated with a better DFS in oropharynx cancer patients (HR = 0.50, 95% CI: 0.33–0.75, *P*=0.001; *I^2^* = 25.8%, *P*=0.260) with no substantial heterogeneity (Figure 3D).

### Sensitivity and publication analysis

We confirmed that there was low heterogeneity in studies in the present meta-analysis by sensitivity analysis. Moreover, no substantial asymmetry was identified by Begg’s tests and funnel plot, in the light of the visual inspection of the shape (Supplementary Figures S1 and S2). This indicated low publication bias and outcomes of meta-analysis to be statistically robust.

## Discussion

The PD-L1/PD-1 pathway has showed a remarkable value among all immune checkpoints because promising and impressing responses in many tumors, such as melanoma, non-small-cell lung cancer (NSCLC) and renal cell carcinoma, were achieved in therapies targeting the PD-1/PD-L1 pathway [33]. In HPV-related carcinomas, antibodies targeting the PD-1/PD-L1 pathway are being assessed in oropharynx cancers, cervical cancer, penile cancer and anal cancer [34–36]. A phase Ib clinical trial demonstrated that the overall response rate (ORR) of squamous cell carcinoma of the head and neck SCC cases receiving pembrolizumab was 18%, and complete response was 5% [37]. The anti-tumor activity of pembrolizumab was also shown in advance cervical cancer, whose ORR was 17% and treatment-related adverse events were acceptable [34]. Recently, a phase II trial of nivolumab in refractory metastatic anal SCC illustrated that the ORR was 21% [35]. Moreover, the clinical studies exploring immunotherapy for the treatment of penile cancer are ongoing [36]. Nevertheless, not all of the cases treated with anti-PD-1/PD-L1 therapy got clinical response. Therefore, it is necessary to identify biomarkers with therapeutic effect to select patients and predict response. PD-L1 expression in tumor cells was considered as a potential response biomarker for PD-1/PD-L1 targeted therapy in most studies, including the clinical trials of nivolumab/pembrolizumab in HNSCC, cervical and anal cancer, despite PD-L1 negative patients could also get clinical benefits from the therapy [7,34,35,37]. Hence, it is necessary to clarify the role of PD-L1 in anogenital and oropharyngeal SCC, which could help to identify more suitable anogenital and oropharyngeal cancer cases for anti-PD-1/PD-L1 therapy.

In the meta-analysis of anogenital SCC, high PD-L1 expression was related to advanced age and higher tumor grade. Furthermore, PD-L1 positive patients held a worse OS in all anogenital SCC, and a worse CSS in penile SCC. PD-L1, as the dominant inhibitory ligand of PD-1, could induce a conformational change of PD1 and weaken T cell-activating signals. Consequently, proliferation, survival, cytokine production and other functions of T cell were inhibited. Additionally, signaling roles of PD-L1 molecule were identified in certain studies. PD-L1 can deliver pro-survival signals to cancer cells, leading to resistance of apoptosis. Furthermore, PD-L1 can prevent tumor cells from immune cytotoxic effects without assistant of PD-1 signal in T cells [33]. Therefore, the anogenital SCC become more aggressive with high PD-L1 expression, such as higher tumor grade, lymph node metastases and worse prognosis.

In HNSCC cells, Interferon-γ (IFN-γ), produced by CD8^+^ tumor-infiltrated lymphocytes (TILs), was demonstrated to control the gene expression of PD-L1 in tumor cells [28]. Higher number of CD8^+^ TILs or TILs were discovered in HPV-positive HNSCC patients compared with HPV-negative cases [28,38]. As a result, accumulation of activated TILs and IFN-γ could explain favorable outcome in PD-L1 positive HNSCC patients, which were HPV positive.

However, IFN-γ, produced by TILs, seems not to be the main reason for the difference of PD-L1 expression between HPV positive and negative anogenital cancer patients, because no significant difference was identified in numbers of CD8^+^ TILs distributing in HPV positive and negative anal and cervical cancer patients [38,39]. In vulvar SCC, there was fewer number of CD8+ TILs in p16 positive cases compared with p16 negative [40]. Therefore, genetic background, including genomic aberrations and aberrant oncogenic signaling may take the primary responsibility for PD-L1 overexpression in anogenital cancers [17,41]. Thus PD-L1 was strongly associated with advanced age, lymph node metastases and worse outcomes in SCC.

In our meta-analysis, there was a distinct relationship between HPV status and PD-L1 expression in oropharyngeal and non-oropharyngeal tumors, especially penile SCC. And it also might explain the relationship between PD-L1 expression and clinical outcome, as HPV-associated oropharyngeal SCC had a better clinical outcome and non-HPV-associated anogenital SCC had a worse clinical outcome. Solomon et al. [38] found that oropharynx SCC patients with high PD-L1 expression seemed to hold a worse OS compared with low PD-L1 expression cases in P16 positive patients, although results were not statistically significant in multivariable analysis (HR = 1.9, 95% CI: 0.7–5.6). In addition to HPV status, the divergent relations between age of patients and PD-L1 expression could also explain that oropharyngeal SCC cases with high PD-L1 expression held a better prognosis, because PD-L1 in oropharynx SCC was more frequent in younger patients.

In order to reduce heterogeneous variables among the anogenital SCC studies, subgroups of meta-analysis were performed based on cancer types, antibody catalogs and cut-off values of PD-L1 positivity. Antibody (clone E1L3N and clone 22C3) and antibody (clone SP142) were reliable to detect PD-L1 expression in anogenital and oropharyngeal SCC, respectively. Moreover, it was rational that cut-off value of PD-L1 positivity was greater than or equal to 5%. Because of limited data of each cancer type in anogenital SCC, we could not perform meta-analysis of each kind of anogenital SCC. However, anogenital SCC (cervical, vaginal, vulvar, penile and anal cancer) had the same embryological origins, as well as unequivocal premalignant and malignant changes existing in entire anogenital region, so we considered that lumping all non-oropharyngeal tumors for meta-analysis was relatively rational.

Undoubtedly, there were some limitations in the meta-analysis. First, relatively fewer vulvar, penile and anal cancer patients were enrolled. Furthermore, no studies investigating the role of PD-L1 in vaginal cancer were found after research. Therefore, clinical studies with higher quality and large sample size are necessary to support our conclusion. Second, chemoradiotherapy also affects expression of PD-L1, but limited studies reported the detailed treatments of patients before examining the PD-L1 expression. As a result, we did not investigate the relationship between various forms of treatments and PD-L1 expression. Third, we estimated HR and 95% CI from Kaplan–Meier curves and with the method described by Altman and Bland [32], which were not accurate as reported by authors. Fourth, we could not judge whether different PD-L1 antibodies could lead to differing results for PD-L1 expression in HPV-related SCC due to the limited data involving antibody catalogs of PD-L1 in HPV-related SCC. Moreover, we should highlight the lack of analytical harmonization to PD-L1 evaluation, such as the consensus for p16 expression associated with HPV.

## Conclusions

The results of the meta-analysis demonstrated that advanced age, higher tumor grade, lymph node metastasis and HPV negativity were associated with high PD-L1 expression in anogenital SCC cases. And anogenital SCC cases that were PD-L1 positive held a worse outcome. For oropharynx cancers, PD-L1 was less frequent in older patients and negative HPV status. Furthermore, PD-L1 expression was related to better prognosis of oropharynx cancer patients. Advanced age and negative HPV status might explain anogenital SCC cases with high PD-L1 expression holding a worse prognosis, compared with oropharynx SCC cases with high PD-L1 expression. Meanwhile, PD-L1 expression should be combined with clinicopathologic features representing high mutation load, including advanced age, higher tumor stage and HPV status, to identify more suitable HPV-related cancer cases for anti-PD-1/PD-L1 therapy.

## Supplementary Material

Supplementary Figures S1-S2 and Tables S1-S3

## Data Availability

All the data used to support the findings of the present study are included within the article. Please contact author for data requests.

## Abbreviations

CI: confidence interval
CSS: cancer-specific survival
DFS: disease-free survival
HPV: human papillomavirus
HR: hazard ratio
IFN: Interferon
IHC: immunohistochemistry
NK: natural killer
OR: odds ratio
ORR: overall response rate
OS: overall survival
PD-1: programmed death 1
PD-L1: programmed death ligand 1
SCC: squamous cell carcinoma
TIL: tumor-infiltrated lymphocyte
