# Supplementary material for: PD-L1 expression in anogenital and oropharyngeal squamous cell carcinomas associated with different clinicopathological features, HPV status and prognosis: a meta-analysis
Source: Biosci Rep. 2021 Mar 26;41(3):BSR20203669. doi: 10.1042/BSR20203669 (PMC8011230; doi:10.1042/BSR20203669)
Supplement: Supplementary Figures S1-S2 and Tables S1-S3 [file BSR-2020-3669_supp.pdf]

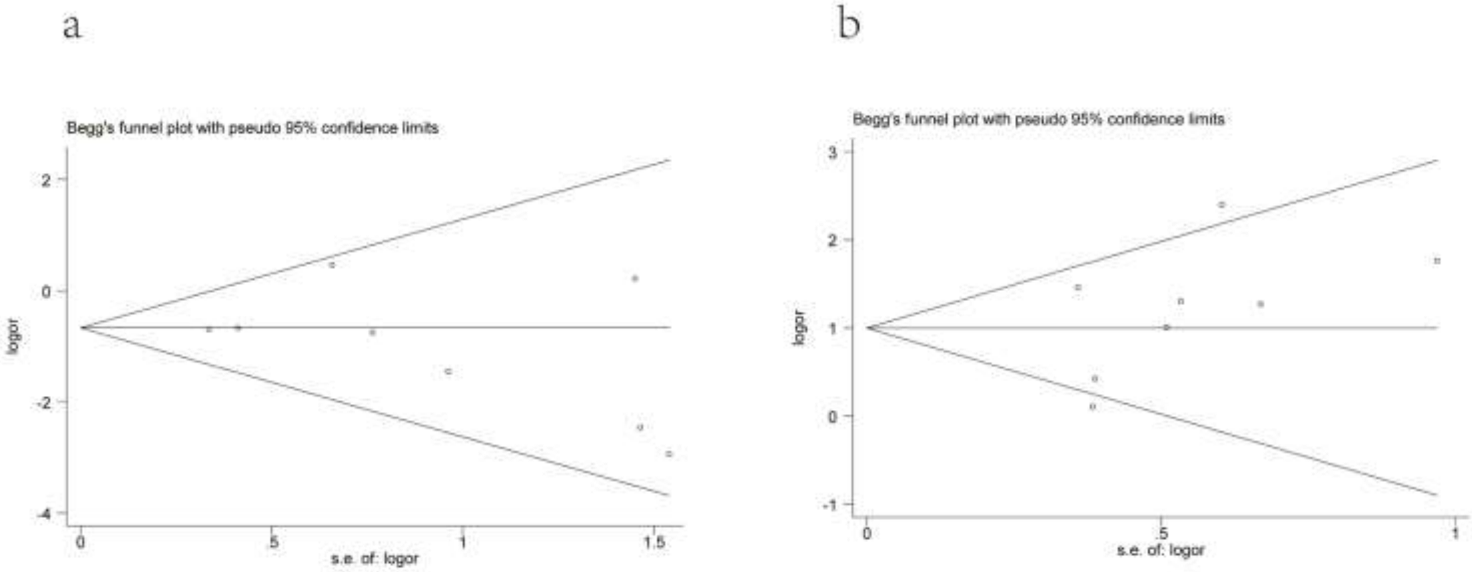

Supplement Figure 1: Funnel plot for the relationship between PD-L1 expression and HPV status in anogenital (a)and oropharyngeal SCC (b).

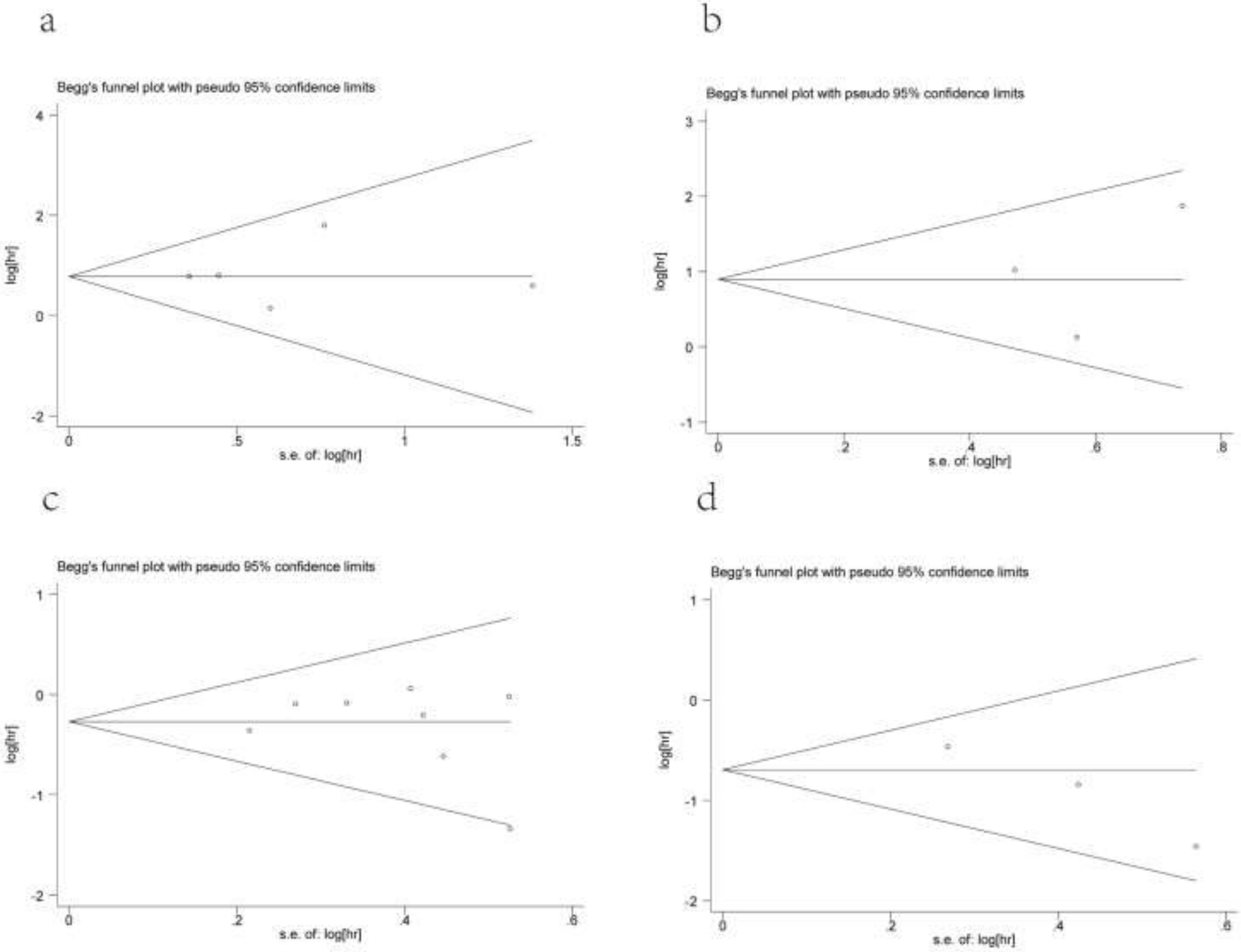
a
Begg's funnel plot with pseudo 95% confidence limits
log[hr]
s.e. of: log[hr]
4
2
0
-2
0
.5
1
1.5
b
Begg's funnel plot with pseudo 95% confidence limits
log[hr]
s.e. of: log[hr]
3
2
1
0
-1
0
.2
.4
.6
.8
c
Begg's funnel plot with pseudo 95% confidence limits
log[hr]
s.e. of: log[hr]
1
0
-1
-2
0
.2
.4
.6
d
Begg's funnel plot with pseudo 95% confidence limits
log[hr]
s.e. of: log[hr]
1
0
-1
-2
0
.2
.4
.6

Supplement Figure 2: Funnel plot for the relationship between PD-L1 expression and oncological prognosis in anogenitaland oropharyngeal SCC. (a) OS of anogenital SCC. (b) CSS of penile SCC. (c) OS of oropharyngeal SCC. (d) DFS of oropharyngeal SCC.

Table S1: Methods for PD-L1 detection

Study	Cancer type	Method	Source	Antibody type	catalog	Antibody dilution
Govindarajan R	anal cancer	IHC	Abcam	rabbit, pAb	NR	NR
Zhao YJ	anal cancer	IHC	Cell Signaling Technology	rabbit, mAb	clone E1L3N	1:100
Heeren AM	cervical cancer	IHC	Cell Signaling Technology	Rabbit, mAb	clone E1L3N	1:200
Wang S	cervical cancer	IHC	Abcam	NR	NR	NR
Udager AM	penile cancer	IHC	E.D. Kwon,Yale University	Mouse, mAb	Clone 5H1	1:500
Deng C	penile cancer	IHC	Cell Signaling Technology	Rabbit, mAb	clone E1L3N	NR
Ottenhof SR	penile cancer	IHC	Cell Signaling Technology	Rabbit, mAb	clone E1L3N	1:200
Howitt BE	vulvar cancer	IHC	NR	Mouse, mAb	clone 9A11	1:100
Sznurkowski JJ	vulvar cancer	IHC	Dako Inc	Mouse, mAb	clone 22C3	NR
Hecking T	vulvar cancer	IHC	Dako Inc	Mouse, mAb	clone 22C3	1:25
Choschzick M	vulvar cancer	IHC	Cell Signaling Technology	Rabbit, mAb	clone E1L3N	1:100
Ukpo OC	oropharynx cancer	IHC	NR	NR	clone A3	1:300
Kim HS	oropharynx cancer	IHC	Dr. Lieping Chen's laboratory	Mouse, mAb	clone 5H1	NR
De Meulenaere A	oropharynx cancer	IHC	Roche	Rabbit, mAb	clone SP142	1:100
Steuer CE	oropharynx cancer	IHC	Cell Signaling Technology	NR	NR	1:20

Fukushima Y	oropharynx cancer	IHC	Ventana	Rabbit, mAb	clone SP142	NR
Hong AM	oropharynx cancer	IHC	Cell Signaling Technology	Rabbit, mAb	clone E1L3N	1:200
Sato F	oropharynx cancer	IHC	Cell Signaling Technology	Rabbit, mAb	clone E1L3N	1:100
Lyford-Pike S	tonsil cancer	IHC	Dr. Lieping Chen's laboratory	Mouse, mAb	clone 5H1	NR
Kwon MJ	tonsil cancer	IHC	Ventana	Rabbit, mAb	clone SP142	1:25

IHC:immunohistochemistry; mAb:Monoclonal antibody; pAb:Monoclonal antibody; NR: not report

Table S2: Detailed result of stratified analysis of association between PDL1 and HPV status in HPV-related SCC.

Analysis	Number of studies (number of patients)	OR(95%CI)	p	model	Heterogeneity	
					I^2 (%)	P_{het}
Anogenital SCC						
Cancer type						
Cervical cancer	1(156)	0.51(0.23-1.14)	0.100			
Anal cancer	2(67)	0.39 (0.08-1.78)	0.224	Fixed	0	0.339
Vulvar cancer	3(181)	0.25(0.02-3.28)	0.294	Random	71.8	0.029
Penile cancer	2(250)	0.49(0.27-0.90)	0.022	Fixed	0	0.946
Antibody catalog						
Clone E1L3N	4(450)	0.55(0.35-0.87)	0.011	Fixed	14.1	0.321
Clone 9A11	1(23)	0.05(0.00-1.08)	0.056			
Clone 22C3	1(103)	0.09(0.00-1.50)	0.093			
Clone 5H1	1(37)	0.47(0.11-2.10)	0.324			
Cut-off value						
≥5%	5(377)	0.50(0.29-0.87)	0.014	Fixed	22.6	0.271
<5%	1(213)	0.50(0.26-0.96)	0.038			
H-score	1(23)	0.05(0.00-1.08)	0.056			
Oropharynx SCC						
Antibody catalog						
Clone E1L3N	1(214)	4.31(2.14-8.71)	0.005			
Clone SP142	3(270)	4.29(2.33-7.91)	0.000	Fixed	39.2	0.193
Clone A3	1(181)	1.11(0.53-2.36)	0.778			
Clone 5H1	2(160)	2.22(0.68-7.26)	0.185	Fixed	39.5	0.198
Cut-off value						
≥5%	5(519)	2.69(1.21-5.97)	0.015	Random	67.6	0.015

<5%	2(306)	4.11(2.30-7.37)	0.000	Fixed	0.0	0.808
H-score	1(97)	3.56(0.96-13.19)	0.058			

SCC: squamous cell carcinoma; OR: odds ratio; CI: confidence intervals

Table S3: Detailed result of stratified analysis of association between PDL1 and prognosis in HPV-related SCC.

Analysis	Number of studies (number of patients)	HR(95%CI)	p	model	Heterogeneity	
					I^2 (%)	P_{het}
Anogenital SCC						
OS						
Cancer type						
Cervical cancer	1(90)	6.07(1.36-26.97)	0.018			
Anal cancer	1(26)	1.82 (0.12-27.33)	0.665			
Vulvar cancer	3(242)	1.96(1.19-3.22)	0.008	Fixed	0	0.628
Antibody catalog						
Clone E1L3N	2(81)	1.25(0.42-3.66)	0.690	Fixed	0	0.765
Clone 22C3	2(187)	2.20(1.27-3.80)	0.005	Fixed	0	0.973
Cut-off value						
≥5%	4(268)	1.96(1.20-3.19)	0.007	Fixed	0	0.817
H-score	1(90)	6.07(1.36-26.97)	0.018			
CSS						
Cancer type						
Penile cancer	3(366)	2.45(1.30-4.65)	0.006	Fixed	45.1	0.162
Antibody catalog						
Clone E1L3N	2(329)	1.94(0.95-3.95)	0.069	Fixed	31.1	0.228
Clone 5H1	1(37)	6.54(1.54-27.79)	0.011			
Cut-off value						
≥5%	2(153)	2.56(0.46-14.44)	0.280	Random	71.5	0.061
<5%	1(213)	2.78(1.10-7.00)	0.030			
Oropharynx SCC						
OS						

Antibody catalog						
Clone E1L3N	2(351)	0.76(0.53-1.08)	0.127	Fixed	0.0	0.489
Clone SP142	3(270)	0.52(0.30-0.91)	0.022	Fixed	36.8	0.205
Clone A3	1(181)	0.91(0.54-1.54)	0.727			
Clone 5H1	1(133)	0.81(0.36-1.86)	0.626			
Cut-off value						
≥5%	5(629)	0.80(0.57-1.11)	0.153	Fixed	21.0	0.281
<5%	2(306)	0.67(0.46-0.98)	0.037	Fixed	0.0	0.606
H-score	1(97)	1.06(0.48-2.35)	0.886			
DFS						
Clone SP142	2(178)	0.34(0.18-0.67)	0.002	Fixed	0	0.386
clone E1L3N	1(137)	0.63(0.37-1.07)	0.085			
Cut-off value						
≥5%	3(315)	0.50(0.33-0.75)	0.001	Fixed	25.8	0.260

SCC: squamous cell carcinoma; OS: overall survival; CSS: cancer specific survival; DFS: disease free survival; HR: hazard ratio; CI: confidence intervals
